# Emotional Design in Chinese Pictographic Character Learning: Effects on Cognitive Load, Aesthetic Pleasure, and Intrinsic Motivation Among CSL Learners

**DOI:** 10.3390/bs16050716

**Published:** 2026-05-07

**Authors:** Bo Liu, Jiaqi Wang, Zhiyang Yue, Jing Ma, Jie Zhang

**Affiliations:** 1 School of Design, Shanghai Jiao Tong University, Shanghai 200240, China; bibobox@sjtu.edu.cn (B.L.); jiaqiwang@sjtu.edu.cn (J.W.); 2 Faculty of Applied Sciences, Macao Polytechnic University, Macao 999078, China; p2320381@mpu.edu.mo (Z.Y.); jpeter.zhang@mpu.edu.mo (J.Z.); 3 School of Art Education, Hubei Institute of Fine Arts, Wuhan 430060, China

**Keywords:** emotional design, Chinese pictographic character learning, anthropomorphism, cognitive load, aesthetic pleasure, intrinsic motivation

## Abstract

The orthographic simplification of Chinese pictographic characters may place a cognitive strain on contemporary learners. Historical evolution has reduced the visual transparency of Chinese pictographic characters, making them increasingly difficult for learners of Chinese as a second language to recognize intuitively. Although current technologies assist in the acquisition of Chinese pictographic characters, how the design of instructional materials shapes learners’ emotional experience in Chinese pictographic character learning remains underexplored. This work, grounded in Emotional Design, designed four versions of instructional materials for 32 Chinese pictographic characters under the title *Revitalize Character*. A within-subjects experiment with 32 learners of Chinese as a second language (CSL) was conducted, employing recognition-based learning tasks, to assess the effects of four conditions: no emotional design (ND), anthropomorphizing emotional design (AD), colorful emotional design (CD), and anthropomorphizing & colorful emotional design (ACD) on cognitive load, aesthetic pleasure, and intrinsic motivation. The experimental findings demonstrate that, in contrast to ND, CD significantly diminished extraneous cognitive load (ECL, p<0.01), and both CD and ACD elevated germane cognitive load (GCL, p<0.01) and markedly improved learners’ aesthetic pleasure (p<0.001). Regarding motivation, AD led to a significantly greater decrease in intrinsic motivation compared to CD (p<0.05). Qualitatively, ACD received more favorable responses from learners than the other conditions, while AD resulted in a subpar learning experience. This study offers empirical evidence for the implementation of emotional design in the recognition-based learning of Chinese pictographic characters, illustrating its potential to improve important aspects of the learning experience.

## 1. Introduction

Chinese pictographic characters, as one of the most significant writing systems globally, have attracted considerable interest among Chinese as a second language (CSL) learners (Fang et al., 2024). Initially, a principal technique for character development entailed the direct depiction of real objects by graphical representations (Hager, 1801). During the process of “clerical change” (Libian), these symbols experienced considerable orthographic reduction, obscuring their original etymological logic in contemporary glyphs (Kwan, 2011). The intricate structure of Chinese character strokes may place demands on the working memory of adult learners, which can constrain their capacity to make strong associations between form and meaning (Kuo et al., 2015). The prevailing trend in adult Chinese character learning resources emphasizes mobile applications (Fang et al., 2024; Qian et al., 2018) and computer-assisted systems (Sun et al., 2024). Interactive technologies now facilitate stroke tracing through Virtual Reality (VR) (Vogel et al., 2022) or the creation of images by selecting radicals (L. Wang et al., 2024). CSL learners have recognized that these digital tools improve engagement in character acquisition (McLaren & Bettinson, 2016).

Nonetheless, these educational tools predominantly emphasize “functional assistance.” The Cognitive-Affective Theory of Multimedia Learning (CATML) posits that although a learner’s emotional state may exert a limited direct effect on academic performance, it significantly affects intrinsic motivation, particularly the motivation to continue engaging with the learning material (Heidig et al., 2015). Research indicates that learners subjected to positive emotional designs regard the materials as less challenging and demonstrate elevated levels of motivation and satisfaction (Um et al., 2012). Investigations by Chou (2009); Tsai et al. (2021) propose that the study of Chinese character acquisition necessitates the amalgamation of subjective factors (such as motivation and emotion) with objective factors (such as learning strategies). While current VR and mobile applications have enhanced learning outcomes via functional support, they frequently neglect the emotional experiences of adult learners confronting complex characters and fail to address issues related to intrinsic motivation. Therefore, there is an urgent necessity to investigate the implementation of emotional design in the context of CSL learners’ character acquisition. This study examines the impact of emotional design on cognitive load, intrinsic motivation, and aesthetic pleasure among CSL learners during character recognition.

### 1.1. Challenges in Chinese Pictographic Character Acquisition for Learners

Since the 1970s, research in applied linguistics has progressively transitioned to a learner-centered paradigm, highlighting the significance of individual affective experiences—such as motivation, attitude, and emotion—as well as positive emotions in the prolonged process of Foreign Language (FL) acquisition (MacIntyre & Mercer, 2014; Tutton & Cohen, 2024; Y. Wang, 2024). The emotions encountered by foreign language learners are deemed to be crucial (L. Zhang & Tsung, 2021). Nonetheless, the pronunciation, spelling, and semantics of Chinese pictographic characters pose considerable difficulties for non-native learners (Shen, 2004). Western learners frequently experience significant negative transfer and learning anxiety when confronted with unfamiliar character structures, owing to the pronounced differences in visual, phonological, and semantic characteristics compared to alphabetic scripts (Schmitt et al., 1994; Tan et al., 2001), which can detrimentally affect their proficiency (Qiao et al., 2025). Moreover, as the quantity of characters to be memorized escalates, the cognitive load may induce weariness and a lack of motivation among learners (Gabbianelli & Formica, 2017, p. 196).

In addressing these issues, academics have created character learning systems to support adult Chinese as a Second Language (CSL) learners. Lan et al. (2009) created a framework to assist learners in mastering essential radical and structural information, applying these concepts to significant scenarios for enhanced understanding. Chen et al. (2019) employed the “keyword-image mnemonic” to facilitate learners’ comprehension of characters. Although these systems have improved cognitive efficiency, their fundamental design is limited to functional implementation, overlooking the active engagement of the learner’s emotional state. To our knowledge, no study has yet examined how emotional design concepts can be employed in adult Chinese pictographic character learning to alleviate boredom. These challenges suggest that adult novice CSL learners may benefit from perceptual and mnemonic support that restores form–meaning connections weakened by historical orthographic change. This study therefore examines the applicability and effects of emotional design in Chinese pictographic character learning.

### 1.2. Emotional Design and Its Affective Impact on Learning Materials

Research in cognitive neuroscience shows that reading Chinese pictographic characters engages neural networks associated with the processing of pictorial representations (Tan et al., 2001). The diminishing pictographic elements in contemporary characters have heightened learning challenges (L. Wang et al., 2024); thus, incorporating images or illustrations as aids can enhance understanding for CSL novices (Liu, 2021). This serves as a gateway to emotional design—the process of reconfiguring visuals in multimedia content to improve visual attractiveness and anthropomorphism (or personification) (Mayer & Estrella, 2014; Plass & Kaplan, 2016).

Emotional design encompasses the application of distinctive, attractive colors to educational components and the use of anthropomorphisms to infuse designs with human traits (Brom et al., 2018; Park et al., 2015). These components augment learner engagement, visual appeal, and motivation (Kaifeng & Pengbo, 2024; Plass & Kaplan, 2016; Schneider et al., 2019). A prevalent technique for generating positive emotional design is the amalgamation of appealing hues with favorable anthropomorphic characteristics, such as human-like facial expressions (X. Wang et al., 2023). Meta-analyses have validated the overall effectiveness of emotional design in knowledge retention and transfer assessments (Brom et al., 2018; R. M. Wong & Adesope, 2021).

Nonetheless, these findings suggest that not all emotional design cues operate through the same mechanism: disagreement persists within the academic community. An eye-tracking study by Park et al. (2015) revealed that although anthropomorphism attracts attention, it does not inherently enhance learning performance. If anthropomorphic aspects function solely as visual embellishments, their impact on learning outcomes is minimal (Slabbert et al., 2022). This points to a functionally important distinction: some emotional design cues may support meaning construction, while others may merely capture attention—a contrast that has yet to be examined in the context of Chinese pictographic character learning.

Furthermore, a meta-analysis by Kaifeng and Pengbo (2024) found that while anthropomorphism significantly improved retention among juvenile learners (standardized mean difference, SMD = 0.42, *p* = 0.001), the effect on adult learners was non-significant (SMD = 0.19, *p* = 0.09), and was associated with significantly elevated extraneous cognitive load (SMD = 0.62, *p* = 0.004), suggesting that decorative anthropomorphic elements may capture initial attention without translating into meaningful learning gains (Starkova et al., 2019). For adult learners in particular, such decorative features may risk being perceived as childish or lacking in professionalism, which could in turn reduce rather than enhance their willingness to engage with the material.

Research has predominantly concentrated on the domains of Science, Technology, Engineering, and Mathematics (STEM) (Bayraktar, 2024). In Technology-Enhanced Learning (TEL), it has been established that the implementation of emotional design enhances active learner engagement (McEvoy & Cowan, 2016). Research on language learning environments, especially regarding Chinese pictographic characters acquisition, is limited. This study seeks to incorporate emotional design into the learning of Chinese pictographic characters, examining its impact on cognitive load, aesthetic experience, and intrinsic motivation among adult learners. Although emotional design shows promise, its effectiveness appears to be context-dependent and may depend on how specific cues are functionally integrated into the learning material.

### 1.3. Leveraging Color Mnemonics in Pictographic Character Instruction

In the *Shuowen Jiezi*, Xu Shen categorized Chinese characters into six classifications (Zhen, 2015), including pictographs (Xiangxing), which are imitative representations of actual objects (Liu, 2021). These characters demonstrate significant visual intuitiveness (A. W.-K. Wong, 2018) and display a “picture superiority effect” when utilized as educational resources. Chinese pictographic characters, in their first development, integrated human aesthetic sensibilities (Chai & Jiang, 2022), offering learners distinct semantic cues (Liu, 2021). Studies have shown that visual mnemonics that integrate visuals and narratives can markedly enhance memory retention for Chinese pictographic characters (Leminen & Bai, 2023; Tsai et al., 2021). This approach draws on dual-coding theory (Paivio, 2010), which posits that human cognition processes verbal and non-verbal (visual) information through parallel channels, and that activating both channels simultaneously can enhance memory encoding (Kurniawan et al., 2022). In the context of Chinese pictographic character instruction, the primary mechanism underlying dual coding is the visual-semantic reconstruction of pictographic meaning, re-establishing the iconic correspondence between a character’s form and its real-world referent. Color plays a supporting role by making the pictorial logic embedded in each character more visually prominent. In the domain of CSL character acquisition, methodologies that integrate visual imagery with semantic representation have proven to be more engaging, beneficial, and user-friendly than stroke-order demonstrations (Chang et al., 2022). This framing positions the study as a comparison between semantically supportive color cues and attention-oriented anthropomorphic cues.

**Figure 1:**
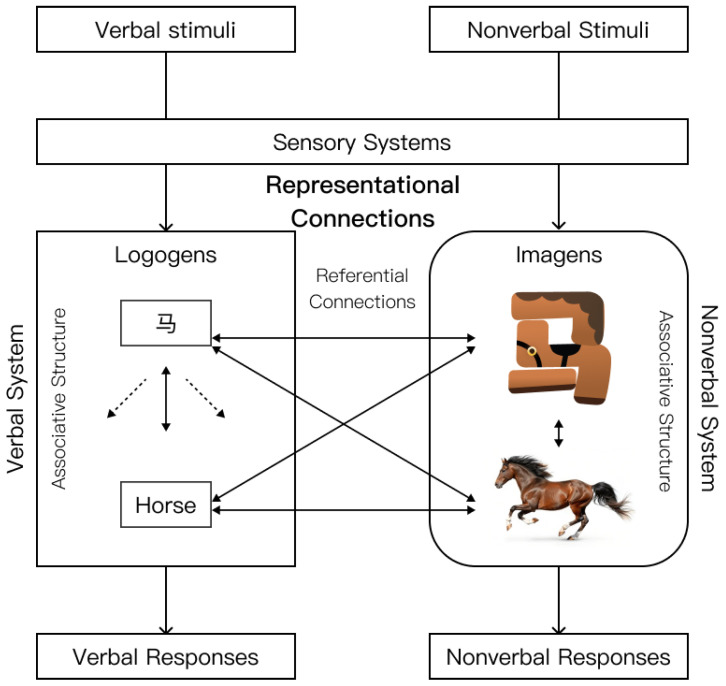
A diagram showing how dual-coding theory and color-coded Chinese pictographic characters can be used. This is based on (Paivio, 1986) and shows how the verbal and nonverbal systems are connected in a way that includes both symbolic and referential links. The arrows indicate representational connections from verbal and nonverbal stimuli to their corresponding systems, referential connections between the verbal and nonverbal systems, and associative connections within each system. In Dual Coding Theory with Characters, the character “马” (horse) is shown in the verbal system as writing and the English word for “horse.” In the non-verbal system, it is shown as a color-coded character and a real-life picture of a horse. The use of color design within the framework of Dual Coding Theory is shown in this picture.

Consequently, we employ color design with the theoretical aim of reducing cognitive strain through this form–meaning mapping. Figure 1 depicts our methodology for implementing dual-coding theory in relation to Chinese pictographic characters. This work took 32 Chinese pictographic characters from *Shuowen Jiezi* and converted them into educational materials based on this design approach. Unlike anthropomorphic cues, which primarily attract visual attention, color in Chinese pictographic character instruction may serve a semantic and mnemonic function by reinforcing the etymological and pictorial logic embedded in each character’s form. The present study therefore compares the effects of functional–semantic color cues against anthropomorphic cues, both independently and in combination, to examine which design approach better supports recognition-based learning among CSL learners.

To address these challenges, this study developed a set of educational resources titled *Revitalize Character* designed to reintegrate etymological logic with modern emotional design principles. The color scheme is not simply decorative but adheres to dual-coding theory (Paivio, 2010), while anthropomorphic features are employed to evoke positive emotions. This dual strategy seeks to diminish cognitive load while augmenting motivation and aesthetic enjoyment from both cognitive and emotional perspectives. This study incorporates emotional design aspects into mnemonic design for adult Chinese learners and examines how these design cues relate to the learning process in this instructional context. Consequently, we propose the subsequent research questions:

- **RQ1:** How should emotional design elements be incorporated into mnemonics for Chinese pictographic characters for adult CSL learners?
- **RQ2:** Do emotional design elements (anthropomorphism and color) significantly affect motivation, aesthetic pleasure, and cognitive load in Chinese pictographic character materials?

To address these questions, we conducted a user study with CSL learners. We quantitatively assessed the influence of various mnemonic designs on learners’ cognitive load, aesthetic pleasure, and intrinsic motivation. We performed semi-structured interviews to thoroughly examine participants’ subjective learning experiences. This study’s findings yield the following key contributions:

- Examined the applicability of CATML-related emotional design principles in the context of Chinese pictographic character learning, with a focus on cognitive load, aesthetic pleasure, intrinsic motivation, and subjective experience rather than direct long-term learning outcomes.
- Provided empirical evidence that isolated anthropomorphic cues may function as decorative interference in adult CSL character learning, informing the balance between learner engagement and perceived professionalism in educational resource design.
- Offered evidence that color and anthropomorphic cues play distinct roles in Chinese pictographic character learning: color may support semantic processing, whereas anthropomorphic features may function more as decorative interference than as learning aids, particularly for adult learners.

## 2. Materials and Methods

### 2.1. Material Design

#### 2.1.1. Stimuli Selection and Classification

Candidate Chinese pictographic characters were first retrieved from the Chinese Orthography Database (COD) and then screened against *Shuowen Jiezi* to confirm pictographic status and etymological descriptions.

The selection process was governed by two primary criteria. First, characters were curated to encompass a diverse range of semantic domains, including nature, animals, the human body, and manmade objects, ensuring referential variety. Second, characters whose pictographic origins were difficult to render visually were excluded. For instance, ‘又’ (right hand) was omitted as its three-fingered depiction might elicit viewer discomfort, while ‘主’ (lamp flame) was discarded because its abstract structure precluded an intuitive visual rendering. To maintain academic rigor, the selection of all characters adhered to the following criteria: (1) they must be pictographs; and (2) their meanings must align with the original etymological definitions in *Shuowen Jiezi*.

The 32 characters were then categorized into four groups based on the type of visual referent depicted in each character’s original glyph form: Human (e.g., 人, 目), Nature (e.g., 山, 水), Animal (e.g., 马, 鸟), and Manmade (e.g., 车, 门). This categorization was adopted to ensure that the stimulus set covered a diverse range of pictographic referents. Table 1 provides a descriptive summary of all 32 characters, including each character’s glyph-form description from *Shuowen Jiezi* and stroke count.

#### 2.1.2. Design Procedure and Visual Manipulation

**Figure 2:**
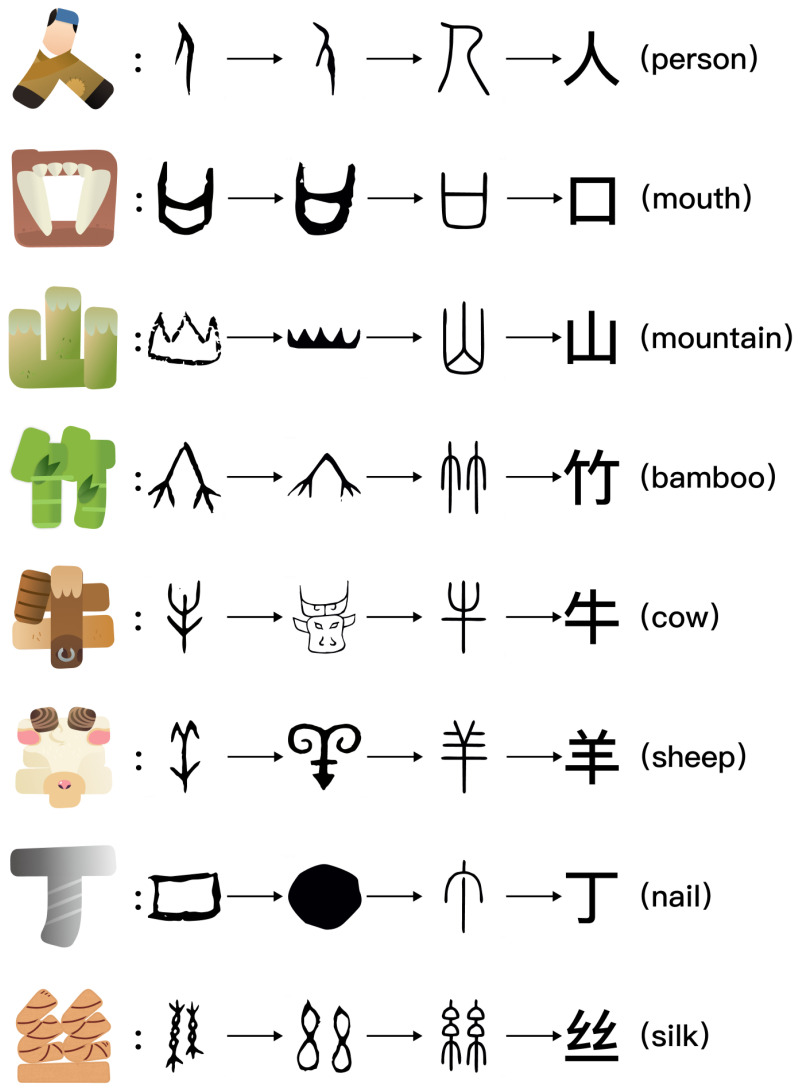
Flowchart depicting the evolution of Chinese pictographic characters. The left section illustrates the design patterns, whereas the right section depicts the evolution process. The sequence of morphological evolution is as follows: Oracle Bone Script, Bronze Inscriptions, Small Seal Script, and Modern Characters. Each row in the picture depicts the progression of an individual character. The evolution of Chinese pictographic characters commences with the Oracle Bone Script, or pictographic form, progressing through stages such as Bronze Inscriptions and Small Seal Script, ultimately resulting in modern simplified or conventional characters. The design preserves the fundamental stroke structure of the characters while integrating visual features pertinent to their semantics. For instance, “人(person)” is depicted with a human-like stance; “口(mouth)” incorporates the representation of teeth; “山(mountain)” reflects the jagged silhouette of mountain peaks; “竹(bamboo)” integrates elements of bamboo stalks and foliage into its strokes; “牛(cow)” and “羊(sheep)” employ symbolic representations of animal horns or forms; “丁(nail)” illustrates the T-shaped structural form of a traditional nail; and “丝(silk)” conveys texture through spiraling lines.

The design process of Chinese pictographic characters adhered rigorously to the character-construction theory outlined in *Shuowen Jiezi* and was categorized into three stages: 1. Feature extraction: examining the progression of Chinese pictographic characters from Oracle Bone Script to contemporary fonts, as seen in Figure 2, to delineate their fundamental morphological and semantic attributes. 2. Morphological restoration: using the character “马” (horse) as an exemplar, the original image was redrafted according to descriptions found in ancient writings (e.g., the character “马” resembles the head, mane, tail, and four legs). 3. Visual enhancement (variable control): To fulfill experimental needs, we implemented two dimensions of visual manipulation. Initially, color was employed to differentiate primary elements; subsequently, anthropomorphism was incorporated. Given the swift human visual recognition of face characteristics (Crouzet & Thorpe, 2011) and the impact of minimalism on cognitive load (Brom et al., 2018), “eyes” were selected as the anthropomorphic component. The specific implementation of each manipulation was as follows.

**Figure 3:**
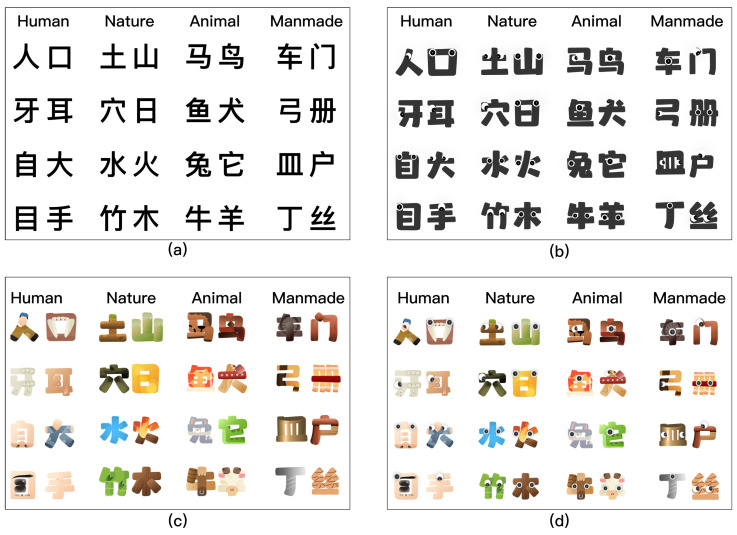
Exhibition of four categories of Chinese pictographic characters educational resources. The illustration depicts four categories of materials labeled (**a**–**d**), corresponding to the categories: Human, Nature, Animal, and Manmade. (**a**) denotes no emotional design (ND); (**b**) denotes anthropomorphizing emotional design (AD) materials; (**c**) denotes colorful emotional design (CD) materials; and (**d**) denotes anthropomorphizing & colorful emotional design (ACD) materials.

For the color manipulation, hues were chosen to match the visual properties of each character’s referent—for example, skin tones for Human characters, green and blue for Nature characters. Nine hues were used in total (skin, black, cream, brown, yellow, blue, red, green, and grey), applied consistently within each semantic category. In this sense, the color manipulation served a dual role: as an emotional design element intended to enhance visual appeal and learner engagement, and as a semantically informative visual scaffold that reinforces the form–meaning connection in each character. For the anthropomorphic manipulation, only eyes were added. This implementation was intentionally minimal, reflecting a single, low-level anthropomorphic cue rather than a comprehensive anthropomorphic treatment. Four eye orientations were used (side-facing, front-facing, upward-facing, and downward-facing), placed according to the spatial structure of each glyph form. Eye orientation varied across characters to accommodate structural differences, which may limit visual uniformity across items (see Figure 3).

All stimulus elements are comprehensively summarized in Table 2, which includes semantic explanations and classifications.

### 2.2. Method

#### 2.2.1. Participants

This study recruited 32 CSL learners eager to learn Chinese pictographic characters (15 females, 17 males) via online recruitment advertisements at a university in China. The participants’ ages varied from 18 to 39 years (M=26.85,SD=4.89). All participants were right-handed, had normal vision, lacked prior experience in learning Chinese pictographic characters, and had been in China for no more than three years (M=13.70,SD=9.97). To increase sample heterogeneity, we deliberately recruited participants from diverse linguistic and cultural backgrounds, including Pakistan, Bangladesh, Poland, Russia, Mongolia, and Colombia.

#### 2.2.2. Experimental Design

This experiment employed a within-subjects design to assess the influence of four types of Chinese pictographic character learning materials on the learning experience. The four types of materials are no emotional design (ND), anthropomorphizing emotional design (AD), colorful emotional design (CD), and anthropomorphizing & colorful emotional design (ACD).

In the experiment, every participant engaged with all four categories of learning materials. A total of 32 Chinese pictographic characters were assigned numerical IDs based on pinyin alphabetical order and divided sequentially into four groups of 8. The character-to-condition assignment was not fixed: it was rotated across four participant groups using a 4 × 4 Latin square design, so that a given character appeared under the ND condition for one group but under a different condition for another. This design simultaneously counterbalanced the order in which participants encountered the four conditions and the pairing of specific characters with specific conditions, ensuring that neither presentation sequence nor item-specific difficulty would systematically confound any single condition. Table 3 illustrates this counterbalancing scheme. Each participant studied four modules comprising four types of materials in varying sequences, with each module containing eight characters grouped into four pairings, each consisting of one character and its accompanying English definition.

Subsequent to the official experiment, all participants submitted feedback regarding their learning experience via a questionnaire. The dependent variables comprised markers of the learning experience, encompassing subjective scale scores and qualitative interview outcomes. A comprehensive account of the experimental approach will be provided in later parts.

#### 2.2.3. Apparatus and Stimuli

**Figure 4:**
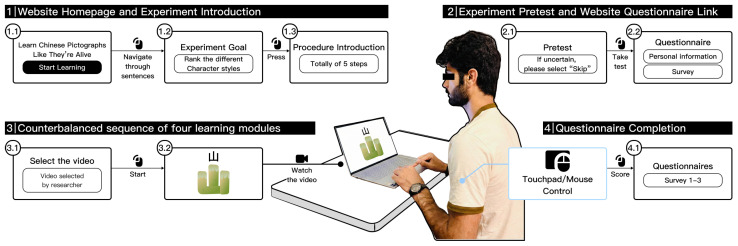
The real experimental environment and the operational flow of the website. The center of the figure shows a photo of the actual experiment setting, where a participant sits in a laboratory environment and performs the task using a laptop, with a touchpad and mouse as the main input devices. Surrounding the participant is the overall operational flow of the experimental website.

**Figure 5:**
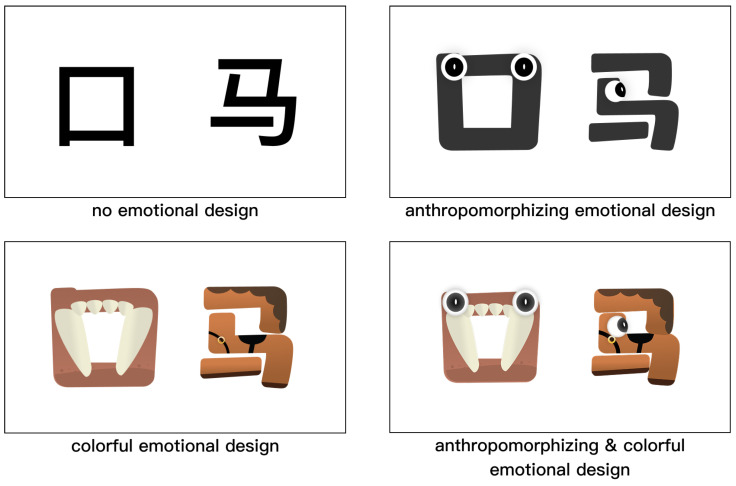
Video screenshots of the four types of Chinese pictographic characters learning materials.

Figure 4 illustrates the experimental apparatus and setting. The laboratory contained desks, seats, and a laptop. The experimental procedure was showcased on a webpage created with Webflow^1^, and videos of the 32 characters were accessed through YouTube links. Figure 4 illustrates a participant viewing the design material. All participants used the same laptop (16-inch screen, 2560 × 1600 resolution) at a typical viewing distance of approximately 40–50 cm. The learning materials were presented as silent videos with no audio component. Video playback was hosted online via YouTube and required a stable internet connection, which was verified before each session. Participants could not pause, rewind, or skip the videos; the presentation pace was fixed by the system. Participants were directed to maintain silence and abstain from any hand motions in the laboratory, concentrating exclusively on the learning materials displayed on the screen. The experimental website offered instructional pages detailing the experimental objectives and procedures to ensure that participants understood the procedure. All educational resources were displayed as static graphics and presented via instructional videos integrated into the website. Throughout the learning process, as illustrated in Figure 5, an exemplar page including the “口-马” (mouth-horse) character pair was presented. Moreover, to regulate the learners’ cognitive load and sustain attention, all films employed character pairs for concise and effective presentation, thereby mitigating distractions resulting from prolonged learning durations.

#### 2.2.4. Data Analysis

Before data analysis, this study conducted checks for data integrity and validity, including missing value detection, outlier identification, and consistency verification of data entry, to ensure the reliability of subsequent statistical inferences. Internal consistency of each multi-item scale was assessed using Cronbach’s alpha. Normality was assessed using the Shapiro–Wilk test (Shapiro & Wilk, 1965), and homogeneity of variance was evaluated using the Levene test. As both assumptions were violated, non-parametric statistical methods were primarily used in the analysis.

For the repeated measures design, the effect of the within-subjects factor “design materials” on multiple cognitive indicators was analyzed using the Friedman non-parametric test (Friedman, 1937). Following the detection of statistically significant effects, post-hoc pairwise comparisons were conducted using the Wilcoxon signed-rank test (Vargha & Delaney, 2000; Wilcoxon, 1992), with Holm-Bonferroni correction (Holm, 1979) applied to adjust for multiple comparisons.

All statistical analyses were performed in the Python (v3.13.5) environment, with the Pingouin package^2^ (Vallat, 2018) used for non-parametric tests, effect size calculations, and multiple comparison adjustments. Data visualization was performed using the Plotnine package^3^ to generate box plots, enhancing the interpretability of the results.

All interviews were audio-recorded and transcribed verbatim. The transcripts were imported into NVivo 14 for coding and analysis. For data analysis, the research team employed thematic analysis (Braun & Clarke, 2006; Terry et al., 2017), using an iterative deductive coding process to analyze the 32 interview transcripts. The first author initially read through all transcripts to achieve data familiarization and generate an initial coding framework. The framework was then systematically applied to all transcripts in NVivo, with continuous modifications and expansions made during the coding process to enhance the comprehensiveness and saturation of the identified themes. Ultimately, four overarching themes encompassing nine sub-themes were derived. To enhance the trustworthiness of the coding, a subset of four transcripts was independently coded by a second researcher using the same codebook in NVivo. The two coders discussed discrepancies and reached consensus through negotiation.

### 2.3. Procedure

**Figure 6:**
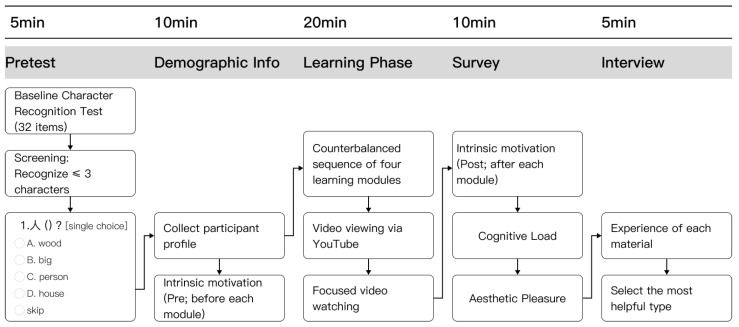
Experimental procedures.

Upon arrival at the laboratory, participants were briefed on the task requirements and signed an informed consent document. The experiment consisted of three phases: the pretest phase, the formal experimental phase (Chinese pictographic characters acquisition), and the evaluation phase. The experimental procedure for each participant is shown in Figure 6.

#### 2.3.1. Pretest

A pretest character recognition task was developed to evaluate the participants’ prior knowledge of the target characters. This task was performed on a laptop utilizing multiple-choice questions. We randomly displayed the 32 original characters, each accompanied by one accurate English definition and three distractors. A “Skip” option was also made available to deter participants from making guesses. Participants were instructed to select the accurate meaning at their own speed.

Following the screening approach of Chang et al. (2014), this study exclusively focused on the character observation task, requiring all participants to be genuine novices. In the pretest, participants who correctly recognized more than three of the 32 target characters were excluded. Pretest accuracy was calculated by dividing the number of correctly recognized characters by 32. The exclusion threshold of three characters corresponds to an accuracy of 9.4%.

#### 2.3.2. Learning Stage

**Figure 7:**
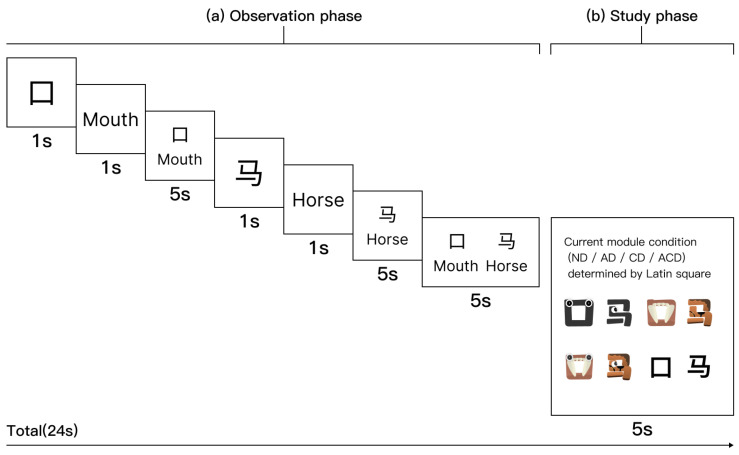
Time flow of the learning task that was being tested. (**a**) Observation phase (19 s): Participants watched as two character pairs, like “口(mouth)” and “马(horse),” were presented, with the characters and their English definitions switching places at set times. (**b**) Study phase (5 s): Participants viewed the version corresponding to the current module condition (ND, AD, CD, or ACD) as determined by the Latin square counterbalancing scheme. Across the experiment, each participant completed four modules and experienced all four conditions.

Figure 7 illustrates the time flow of a representative learning module. The full learning phase comprised four modules, one under each design condition (ND, AD, CD, and ACD), together covering all 32 Chinese pictographic characters arranged into 16 character pairs. Each module contained 8 characters arranged into 4 pairs and consisted of three consecutive rounds. In each round, the four character pairs in the current module were presented once (24 s per pair), yielding approximately 4.8 min per module and approximately 19 min for the full learning phase across all four modules. Participants were permitted to pause and rest between modules. The learning workflow had two stages: (a) Observation phase. This phase endured for 19 s and sought to direct participants’ focus towards the structure and significance of the characters. The presentation approach involved displaying the first character of the pair for 1 s in its original form, followed by its English meaning for 1 s, and then presenting both simultaneously for 5 s; the second character was exhibited using the same procedure. Ultimately, both the original characters and their English definitions were presented concurrently for 5 s to enhance retention. (b) Study phase. Subsequent to the observation phase, a 5-s study period commenced. During this period, participants viewed the version of the learning material corresponding to the current module condition, as determined by the Latin square counterbalancing scheme.

#### 2.3.3. Evaluation Stage

For each design condition, intrinsic motivation was measured immediately before and immediately after the module using the same scale by Guay et al. (2000). Thus, each participant provided four baseline scores and four post-learning scores, yielding four condition-specific change scores (baseline − post), one for each design condition.

### 2.4. Data Collection

Both quantitative and qualitative data were gathered to rigorously assess the influence of various character design materials on the learning experience.

**Cognitive Load.** For each design condition, participants rated cognitive load using the questionnaire by Leppink et al. (2013), which measures three dimensions: Intrinsic Cognitive Load (ICL; α = 0.914; e.g., “This learning material covers topics that are very complex.”), Extraneous Cognitive Load (ECL; α = 0.902; e.g., “During the study, the design and explanations of this learning material were very unclear”), and Germane Cognitive Load (GCL; α = 0.935; e.g., “This learning material really enhanced my understanding of the topics covered.”). A 0–10 Likert scale was used (0 = not at all, 10 = absolutely agree). Higher scores indicate greater perceived cognitive load.

**Aesthetic Pleasure.** For each design condition, participants rated aesthetic perceptions using the visual appeal subscale (5 items) of the design enjoyment scale (Blijlevens et al., 2017). This scale has five items assessing aesthetic appeal, attractiveness, and enjoyment (α = 0.978; e.g., “I find this design visually appealing”). A 7-point Likert scale was used (1 = strongly disagree, 7 = strongly agree). Higher scores indicate more favorable aesthetic perceptions.

**Intrinsic Motivation.** Intrinsic motivation was measured using the Situational Interest and Motivation Scale (Guay et al., 2000), comprising four items (α = 0.966; e.g., “Because I think that this material is interesting”). The same four items were administered immediately before and after each learning module. For each participant, this yielded four condition-specific baseline − post difference scores. A 7-point Likert scale was used (1 = strongly disagree, 7 = strongly agree). Higher scores indicate stronger intrinsic motivation; a positive change score (baseline − post) indicates decreased motivation after learning, while a negative change score indicates increased motivation.

**Interview.** Upon completion of all exercises, we conducted semi-structured interviews with each participant to obtain a more profound insight into their subjective emotions and impressions regarding the various design materials. All interviews were documented and focused on the subsequent two principal questions: 1. What particular emotions or experiences did you have while utilizing various design elements to learn Chinese pictographic characters? 2. Which design material did you prefer the most? What is the reason?

## 3. Results

### 3.1. Cognitive Load

**Figure 8:**
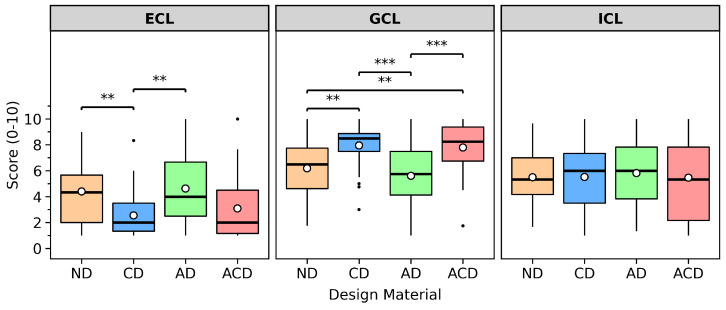
Comparison of intrinsic cognitive load (ICL), extraneous cognitive load (ECL), and germane cognitive load (GCL) across different design materials (ND, CD, AD, and ACD) during Chinese pictographic character learning tasks. The white dots indicate mean values, and the black dots indicate outliers. ** = *p* < 0.01, *** = *p* < 0.001.

As shown in Figure 8, this study used the germane cognitive load (GCL) subscale as the operational indicator of learners’ constructive cognitive processing. The three cognitive load subscales exhibited good internal consistency, with Cronbach’s α values of ICL = 0.914, ECL = 0.902, and GCL = 0.935, respectively. Descriptive statistics are presented as medians and interquartile ranges, denoted as Mdn [IQR]. The Mdn [IQR] for the four design materials regarding intrinsic cognitive load (ICL), extraneous cognitive load (ECL), and germane cognitive load (GCL) were as follows: ICL, ND = 5.33 [2.83], CD = 6.00 [3.83], AD = 6.00 [4.00], ACD = 5.33 [5.67]; ECL, ND = 4.33 [3.67], CD = 2.00 [2.17], AD = 4.00 [4.17], ACD = 2.00 [3.33]; GCL, ND = 6.50 [3.13], CD = 8.50 [1.38], AD = 5.75 [3.38], ACD = 8.25 [2.63].

For ICL, the Friedman test revealed no statistically significant differences across conditions ($\chi_{F}^{2}$ = 0.447, *p* = 0.931).

Regarding extraneous cognitive load (ECL), the Friedman test revealed a significant main effect of material type ($\chi_{F}^{2}=20.892$, p<0.001). Wilcoxon signed-rank tests were further conducted for pairwise comparisons, with Holm’s method applied to correct for multiple comparisons. Results indicated that the median ECL in the ND condition was higher than in the CD condition (ND: Mdn=4.33 [3.67]; CD: Mdn=2.00 [2.17]), and this difference reached statistical significance after Holm correction (p=0.0032). Similarly, the median ECL in the AD condition was also higher than in the CD condition (AD: Mdn=4.00 [4.17]; CD: Mdn=2.00 [2.17]), and this difference was statistically significant after Holm correction (p=0.0031). No other pairwise comparisons reached significance.

For germane cognitive load (GCL), the Friedman test also showed significant differences ($\chi_{F}^{2}=27.650$, p<0.001). Holm-corrected Wilcoxon post-hoc comparisons indicated that the median GCL in the CD condition was higher than in the ND condition (CD: Mdn=8.50 [1.38]; ND: Mdn=6.50 [3.13]), with the difference being statistically significant after Holm correction (p=0.0024); the median GCL in the CD condition was also significantly higher than in the AD condition (CD: Mdn=8.50 [1.38]; AD: Mdn=5.75 [3.38], p<0.001). Additionally, the median GCL in the ACD condition was higher than in the ND condition (ACD: Mdn=8.25 [2.63]; ND: Mdn=6.50 [3.13]), and this difference reached statistical significance after Holm correction (p=0.0060); the median GCL in the ACD condition was also significantly higher than in the AD condition (ACD: Mdn=8.25 [2.63]; AD: Mdn=5.75 [3.38], p<0.001). The difference between CD and ACD did not reach statistical significance.

In summary, significant differences were found across different design materials in terms of extraneous cognitive load and germane cognitive load, while no statistically significant differences were detected in intrinsic cognitive load. Regarding the significant pairwise comparisons, the extraneous cognitive load in the CD condition was lower than in the ND and AD conditions; for the germane cognitive load indicator, the medians in the CD and ACD conditions were higher than those in some of the comparison groups (ND and AD).

### 3.2. Aesthetic Pleasure

**Figure 9:**
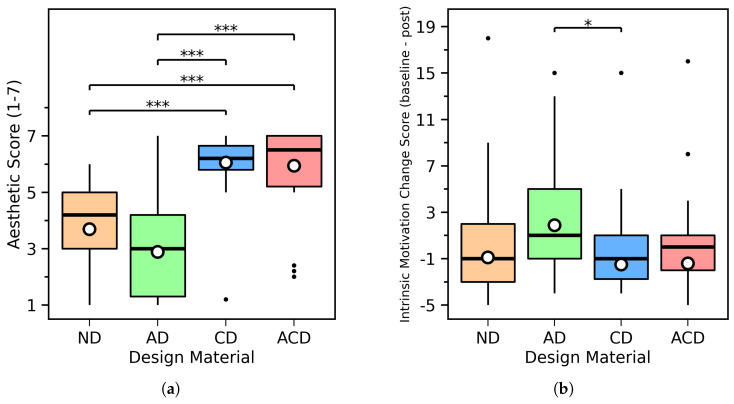
Comparison of two user evaluation indicators. (**a**) Aesthetic scores (1–7); (**b**) Intrinsic motivation change scores (baseline − post). Positive values indicate a decrease in intrinsic motivation after learning, whereas negative values indicate an increase. The white dots indicate mean values, and the black dots indicate outliers. * = *p* < 0.05, *** = *p* < 0.001.

As shown in Figure 9a, the aesthetic pleasure scale exhibited good internal consistency, with Cronbach’s α=0.978. Descriptive statistics for this metric are presented as medians and interquartile ranges, denoted as Mdn [IQR]. The Mdn [IQR] for the four design materials on this metric were as follows: ND = 4.2 [2.05], AD = 2.8 [2.90], CD = 6.2 [0.85], and ACD = 6.5 [1.80]. A Friedman test revealed significant differences in aesthetic pleasure across the four design conditions ($\chi_{F}^{2}=69.899,\;p<0.001$).

Wilcoxon signed-rank tests were further conducted for pairwise comparisons, with the Bonferroni method applied to correct for multiple comparisons. Results showed that the median aesthetic rating in the CD condition was higher than in the ND condition (CD: Mdn=6.2 [0.85]; ND: Mdn=4.2 [2.05]), and this difference reached statistical significance after Bonferroni correction (p<0.001). The median aesthetic rating in the ACD condition was also higher than in the ND condition (ACD: Mdn=6.5 [1.80]; ND: Mdn=4.2 [2.05]), and this difference was statistically significant after Bonferroni correction (p<0.001). Similarly, the median aesthetic rating in the CD condition was higher than in the AD condition (CD: Mdn=6.2 [0.85]; AD: Mdn=2.8 [2.90]), which was statistically significant after Bonferroni correction (p<0.001); the median aesthetic rating in the ACD condition was also higher than in the AD condition (ACD: Mdn=6.5 [1.80]; AD: Mdn=2.8 [2.90]), and this difference reached statistical significance after Bonferroni correction (adjusted p<0.001). The difference between ND and AD did not reach statistical significance (p=0.265), nor did the difference between CD and ACD (p=0.746).

In summary, aesthetic pleasure ratings were significantly higher in CD than in ND and AD, and were also significantly higher in ACD than in ND and AD. No statistically significant difference was detected between CD and ACD.

### 3.3. Intrinsic Motivation

As shown in Figure 9b, the intrinsic motivation change score is defined as the baseline score minus the post-learning score (baseline − post); thus, positive values indicate that post-learning intrinsic motivation was lower than at baseline (i.e., a decrease in motivation), while negative values indicate that post-learning intrinsic motivation was higher than at baseline (i.e., an increase in motivation). The post-learning intrinsic motivation scale exhibited good internal consistency, with Cronbach’s α=0.966. Descriptive statistics for this metric are presented as medians and interquartile ranges, denoted as Mdn [IQR]. The Mdn [IQR] across the four design material conditions were as follows: ND = −2.0 [5.5], CD = −2.0 [4.5], AD = 1.0 [7.0], and ACD = −1.0 [4.5]. A Friedman test revealed a significant difference in intrinsic motivation change scores across the four design conditions ($\chi_{F}^{2}=9.219,\;p=0.0265$).

Subsequently, post-hoc comparisons were conducted using Wilcoxon signed-rank tests, with Holm’s method applied to correct for multiple comparisons. Results showed that only the difference between CD and AD reached statistical significance: the median change score in the AD condition was higher than in the CD condition (AD: Mdn=1.0 [7.0]; CD: Mdn=−2.0 [4.5]), and this difference remained statistically significant after Holm correction (adjusted p=0.0233). No other pairwise comparisons reached significance (ND vs. CD: p=1.000; ND vs. AD: p=0.135; ND vs. ACD: p=1.000; CD vs. ACD: p=1.000; AD vs. ACD: p=0.051).

Under the baseline − post definition used in this study, only the AD–CD contrast reached statistical significance.

### 3.4. Interview Results

To complement the quantitative findings and gain a deeper understanding of participants’ subjective experiences, preferences, and reasoning behind their responses, semi-structured interviews were conducted. While the quantitative measures captured the magnitude and direction of differences across design conditions, the qualitative data aimed to illuminate why participants responded as they did and how they experienced the different learning materials. Thematic analysis of the 32 interview transcripts yielded four overarching themes and nine sub-themes, as summarized in Table 4. The following sections present each theme with representative excerpts.

Concerning choices for learning materials, ACD garnered the most favorable feedback (17/32). Participants (P7, 18, 27) concurred that ACD significantly alleviated the cognitive load and rendered abstract Chinese pictographic characters “narrative” and “captivating.” Twelve participants preferred CD; individuals such as P12 and P14 highlighted that CD facilitated associations between distinct pictures and tangible items, aiding their rapid comprehension of the characters’ meanings. Only 3 participants (3/32) preferred ND, specifically P20, P22, and P29, who remarked that the ND materials were clear and straightforward. No participants favored the AD materials.

Notably, although no participants favored AD, ACD—which shares the same eye-based anthropomorphic feature—received the highest preference (17/32). Participants often criticized isolated eye-based anthropomorphism in AD as distracting or unattractive; however, when combined with color and semantic cues in ACD, the same feature was more often perceived as lively or engaging. This suggests that anthropomorphic elements alone may not enhance appeal, but their integration with color and semantic cues can transform the perceived effect of the same design feature.

#### 3.4.1. Visual Appeal and Novelty

In the interviews, the visual monotony of the ND group was a concern raised by several participants (P1, P6, P10, P13). Several participants stated that the original Chinese pictographic characters, as learning materials, lacked appeal and appeared dull or uninteresting. For example, one participant noted: “This design material does not have any like design, it’s really boring.” (ND) (P1). Another participant pointed out: “It’s just the basic and traditional one. This is too plain.” (ND) (P6, P10, P13). These responses suggest that some participants perceived the original characters as lacking innovation and variety, which may contribute to learning fatigue. In contrast, ACD and CD exhibited high levels of appeal, effectively capturing participants’ attention. Participants such as P16 mentioned: “This is colorful, and they are designed in a way that gives a clean and tidy message.” (ACD) (P16, P27). However, AD was perceived as lacking attraction; specifically, the combination of black-and-white characters with eye patterns failed to produce the intended visual effect. For instance: “But this one only has eyes, and only in black color. It is not attractive.” (AD) (P13).

#### 3.4.2. Clarity of Design

Feedback regarding design clarity was diverse, with some participants favoring the clear style of the ND group. P30 stated: “This is also good because it’s clear, it’s visible, and clearly seen.” (ND). Additionally, ND was characterized by P22 as “simple and professional.” However, the design of anthropomorphized characters was perceived by some participants as overly complex and difficult to understand: “The design feels thick, complex, and difficult to read, making it hard to follow.” (AD) (P20, P21). Other participants noted that imbuing characters with human features actually increased confusion: “It was a little bit confusing, like it was relating to some living material.” (AD) (P31). Furthermore, P22 pointed out that the excessively thick lines in CD affected the clarity of the characters: “I find the strokes of the character hard to see clearly because they are too thick.” (CD) (P22). These observations highlight the importance of maintaining visual clarity in Chinese pictographic characters instructional materials.

#### 3.4.3. Comprehensibility of Design

Regarding the comprehensibility of the designs, the lack of effective instructional aids in the ND group was identified by several participants (P3, P5, P10, P32) as a notable concern. Participants such as P3 expressed that the original characters failed to provide useful learning cues or assistance: “Very difficult to understand and offers no clue or aid for learning, making it unhelpful for memorization.” Consequently, the symbolic design elements in CD and ACD provided effective cognitive scaffolds, enhancing comprehensibility. For example: “When design elements match the meaning, they help connect concepts, aid memory, and make learning quicker.” (CD & ACD) (P7, P14, P26, P27, P30). P19 mentioned: “It’s attractive because we can understand—it gives a kind of explanation.” (ACD). This feedback suggests that symbolic and semantic aids within character design elements may help learners grasp the content more effectively.

#### 3.4.4. Impact of Eye Design

Concerning the addition of eye patterns in AD, feedback from several participants indicated that this design element could produce both positive and negative effects. Some participants felt that the eye design in ACD added a sense of liveliness to the characters and helped express emotions: “Eyes make characters appear lively, engaging, and sometimes help illustrate meaning.” (ACD) (P13, P30). However, others pointed out that eye designs could be distracting or even make the characters appear too human-like, thereby hindering the learning experience: “Eye designs can feel distracting or too humanlike, making it harder to focus or understand.” (AD & ACD) (P6, P12, P17). These responses suggest that while eye designs may enhance interactivity, their effectiveness appears to depend on integration with other design elements such as color schemes and symbolic cues.

Taken together, the quantitative and qualitative findings largely converge. Both data sources indicate that CD and ACD outperformed ND and AD in terms of aesthetic appeal and learning experience, while AD consistently received the least favorable evaluations. The qualitative data further contextualized these patterns: participants described isolated anthropomorphic features in AD as distracting and unattractive, which helps explain the higher extraneous cognitive load and greater decline in intrinsic motivation observed in the quantitative results. However, some nuances emerged. Although the quantitative analysis revealed no statistically significant difference in aesthetic pleasure between CD and ACD, ACD was the most preferred design in the interviews (17 out of 32 participants), suggesting that the integration of anthropomorphic elements with color and semantic cues created a qualitative appeal that standardized scales did not fully capture.

## 4. Discussion

This study examined the effects of four character design approaches (ND, AD, CD, ACD) on cognitive load, aesthetic pleasure, and intrinsic motivation in Chinese pictographic character learning. The quantitative results revealed that CD and ACD were associated with lower extraneous cognitive load, higher germane cognitive load, and greater aesthetic pleasure compared to ND and AD, while AD showed the greatest decline in intrinsic motivation. The qualitative findings largely converged with these patterns: participants criticized ND as visually monotonous and AD as distracting, whereas ACD received the highest overall preference (17 out of 32 participants), suggesting that the combination of color, symbolic cues, and anthropomorphic features was perceived as particularly appealing by participants.

### 4.1. RQ1. How Should Emotional Design Elements Be Incorporated into Mnemonics for Chinese Pictographic Characters for Adult CSL Learners?

Concerning RQ1, the findings suggest that emotional design elements should be incorporated in ways that prioritize semantic support and visual clarity, rather than being treated as decorative additions. Quantitatively, CD showed the clearest advantage in reducing extraneous cognitive load, while both CD and ACD were associated with higher germane cognitive load and aesthetic pleasure than ND and AD. Qualitatively, ACD received the highest overall preference in the interviews, suggesting that anthropomorphic cues may be more appealing when integrated with meaningful color-based cues rather than used in isolation. These findings are consistent with the application of Dual Coding Theory to pictographic writing. This theory posits that human cognition depends on the simultaneous processing of verbal and non-verbal (visual) systems (Paivio, 2010). The findings of this study align closely with the hypothesis posited by Shen (2010) that Chinese vocabulary facilitates dual coding. Furthermore, it should be noted that many pictographs serve as radicals or semantic components within more complex types of Chinese characters (such as compound ideographs and phono-semantic compounds). Associative compounds, for instance, are characters formed by combining individual pictographic symbols into one or more units to express commonalities or suggest specific associations (X. Zhang et al., 2022). This suggests that the emotional design effects observed in the current study may have broader relevance for Chinese character learning, extending beyond pictographs themselves.

During the protracted “clerical change” (Libian), Chinese pictographic characters experienced significant glyph simplification. This resulted in a diminished visual association between contemporary glyphs and their original etymological significance, which may be one contributing factor to the elevated cognitive load experienced by adult learners (Kuo et al., 2015; Kwan, 2011). The *Revitalize Character* materials developed in this study offer learners distinct non-verbal representations (Imagens) by enhancing color and form, thereby establishing a direct connection with the semantic meanings in the verbal system (Logogens) and compensating for the deficiency in visual intuitiveness. Conversely, in the present study, isolated anthropomorphic features such as the eye-based design in the AD condition tended to be perceived as ”meaningless decorations” (Slabbert et al., 2022), possibly due to their lack of semantic integration, which may have increased cognitive strain by diverting attention. However, this interpretation is based on a single visual manipulation and may not generalize to all forms of anthropomorphic design.

This illustrates that in the creation of mnemonic materials for Chinese pictographic characters aimed at adults, emotional components should not be superficial embellishments. They should function as semantically informative visual scaffolds—design elements that carry meaning relevant to the character’s etymology—thereby aiding learners in forming cognitive links between abstract symbols and tangible pictorial representations.

The present findings can be understood in relation to discussions of extraneous processing and decorative interference in multimedia learning. Prior research suggests that visual elements with low relevance to the instructional goal—especially seductive graphics—may create extraneous processing, that is, cognitive processing that does not support the instructional goal (Mayer, 2005; Sung & Mayer, 2012). Research on seductive details and subsequent meta-analytic evidence also indicates that interesting but irrelevant additions can interfere with learning, although their effects may vary depending on design and contextual factors (Harp & Mayer, 1998; Sundararajan & Adesope, 2020). At the same time, the work of Schneider et al. (2016) on decorative pictures and emotional design suggests that decorative pictures should not be understood only as sources of interference, since their effects may also depend on emotional valence and context-relatedness.

In the present context, this discussion helps explain the different roles of color cues and eye-based anthropomorphic cues. The key issue is not simply whether a visual element is emotional or decorative, but whether it forms a semantic connection with the specific learning content. The color cues in the CD condition were connected to the characters’ etymological meanings and form–meaning relations, making them closer to learning-relevant visual cues. By contrast, the isolated eye-based anthropomorphic cues in the AD condition lacked such stable semantic connections and were therefore more likely to function as decorative interference: they could attract attention but did not necessarily support meaning construction. The ACD condition suggests that when eye-based anthropomorphic cues are presented together with color cues and meaning-oriented visual information, they may become more acceptable and engaging rather than functioning as isolated embellishments.

Therefore, in CSL pictographic character learning materials, the value of emotional design elements appears to depend not merely on visual attractiveness, but also on their semantic connection to the characters’ etymological meanings and form–meaning relations.

### 4.2. RQ2. Do Emotional Design Elements (Anthropomorphism and Color) Significantly Affect Motivation, Aesthetic Pleasure, and Cognitive Load in Chinese Pictographic Character Materials?

Concerning RQ2, the anthropomorphizing emotional design (AD) was not effective in alleviating extraneous cognitive strain in this sample. Within the scope of this study—adult learners, pictographic characters, and minimal eye-based anthropomorphism—these findings are consistent with the notion of “decorative interference” in multimedia learning (Slabbert et al., 2022), suggesting that the critical factor may not be ornamentation per se, but whether design elements are instructionally meaningful and semantically integrated. Although eye-based anthropomorphic cues may increase visual interest in some cases, the isolated eye-based cues in the AD condition did not show favorable effects on the learner-experience indicators measured in this study. When the eye-based anthropomorphic cues used here served solely as visual embellishments and did not align with the characters’ semantics, they may instead have acted as a cognitive distraction. Participants (P3, P4) indicated in the interviews that such designs elicited feelings of “confusion, strangeness, and difficulty in comprehension.”

Moreover, in this sample, adult learners (P20, P22, P29) expressed elevated expectations for “professionalism.” In this study, professionalism refers to visual clarity, legibility, and perceived educational appropriateness. For these learners, excessive ornamentation devoid of functional support was regarded as “childish” or a “source of confusion.” This suggests that the implementation of emotional design in the acquisition of Chinese pictographic characters may need to balance “engagement” with “rigor”.

The more favorable evaluations of the ACD condition suggest that eye-based anthropomorphism alone was insufficient and potentially distracting, but when embedded within a color-supported, meaning-oriented design, it became more acceptable and engaging. This pattern indicates that the acceptability and possible cognitive value of eye-based anthropomorphic cues may depend on whether they are semantically integrated with other design features, such as color schemes with referential functions, while maintaining visual clarity and educational appropriateness.

## 5. Limitations and Future Work

This study examined the effects of anthropomorphism and color design in recognition-based learning of Chinese pictographic characters; however, several limitations remain.

**Measurement scope.** A key limitation is that the paper’s claims concern learning support, yet no delayed retention test was administered to assess longer-term learning outcomes. The present findings are therefore limited to immediate effects. Despite this, the identified emotional regulating mechanisms establish a basis for future investigations into long-term cognitive strategies. Future studies should integrate learning outcomes to more thoroughly elucidate the influence of anthropomorphism and color design on the retention and transfer of Chinese pictographic characters acquisition. Additionally, all participants viewed the original characters with English meanings before encountering condition-specific materials. This shared baseline exposure was pedagogically motivated but may have partially attenuated observable differences between design conditions. Characters were also presented in fixed pairs based on numerical order rather than semantic relatedness, with no control for potential pair-specific effects on memorability. Future studies could also explicitly model such item- and stimulus-level variability through mixed-effects approaches.

**Stimulus scope.** The sample of Chinese pictographic characters utilized in the study did not encompass all categories of pictographs, thereby impacting the generalizability of the findings. This study employs a cartoonish design style, which enhances learning interest to a degree; however, its long-term suitability for adult learners remains unknown. Future research should compare different visual styles ranging from minimal-semantic to more playful or cartoonized designs to determine which approaches best serve adult learners. Moreover, the study employed a short-term recognition-oriented task rather than a broader character learning paradigm encompassing writing, recall, or long-term retention. Future research should broaden the scope of character samples, adopt more comprehensive learning tasks, and analyze the disparities in acceptance and learning outcomes across other age groups to strengthen the validity of the conclusions. Furthermore, the character subsets were grouped by pinyin alphabetical order, and the selection prioritized pictographic transparency rather than controlled matching on difficulty-related properties such as stroke count or structural complexity. Although the Latin square rotation ensured that each character appeared under each condition equally often across the full sample, observed differences between conditions could partly reflect item-specific properties at the individual level. Future studies should consider explicitly matching character subsets on difficulty-related features to more effectively isolate condition effects.

**Sample and generalizability.** The within-subject sample size (*n* = 32) was adequate for this exploratory controlled design, but the modest sample limits broader generalization. Certain individuals may have had varying degrees of incidental exposure to Chinese pictographic characters, which could have resulted in proficiency discrepancies. Future studies could address these issues by utilizing larger sample sizes and implementing more rigorous screening protocols. In addition, all outcome measures in the present study were based on self-report scales, and no psychophysiological indicators of cognitive effort (e.g., eye-tracking, electroencephalography (EEG), or galvanic skin response) were collected. Future studies could combine subjective and physiological measures to provide more convergent evidence.

**Cultural and design interpretation.** The present sample, while internationally diverse, was not designed to test cultural variation systematically. It remains an open question whether variables such as aesthetic preferences, receptivity to anthropomorphic features, and tolerance for cartoonish styles vary across cultural backgrounds. Future studies could test whether these variables differ systematically by recruiting culturally defined subsamples of sufficient size for between-group comparisons.

## 6. Conclusions

This study developed and assessed *Revitalize Character*, a set of instructional materials based on Chinese pictographic characters that incorporate emotional design techniques (color and anthropomorphism). By examining the etymological roots of Chinese pictographic characters and applying Dual Coding Theory, we evaluated the effects of these design materials on learner-experience variables including cognitive load, aesthetic pleasure, and intrinsic motivation. The results demonstrate that for learners of Chinese as a Second Language (CSL), colorful emotional design (CD) and combined anthropomorphizing and colorful emotional design (ACD) enhance both aesthetic pleasure and germane cognitive load (GCL) relative to materials with no emotional design (ND). Specifically, CD significantly reduced extraneous cognitive load (ECL) compared to ND; while the ACD group also exhibited lower mean ECL scores, this reduction did not reach statistical significance. Qualitative interviews further indicated that ACD materials yielded the most favorable subjective user experience. Conversely, the study suggests that isolated eye-based anthropomorphic design elements may negatively impact the learning experience. Intrinsic motivation declined more in the AD condition than in the CD condition. This study provides context-specific empirical evidence regarding how color and anthropomorphic cues shape learner experience, cognitive load, and short-term recognition-oriented learning in Chinese pictographic character materials. Rather than proposing a new theoretical framework, it examines the applicability of existing emotional design and dual-coding perspectives in this instructional context. Overall, a key principle emerges from this work: semantically meaningful color-based design appears more consistently beneficial than eye-based anthropomorphic decoration alone, while integrated combinations of both may improve subjective appeal if legibility and clarity are maintained. These findings concern learner-experience variables in a short-term recognition-oriented task and should not be interpreted as direct evidence of improved retention, recall, or transfer.

## Figures and Tables

**Table 1:** Descriptive summary of the 32 Chinese pictographic characters used in the study.

No.	Character	*Shuowen Jiezi* Description	Stroke Count
1	册	Depicts bamboo slips of varying lengths bound with two cords	5
2	车	Depicts the form of a wheeled vehicle	4
3	大	Depicts a person with arms outstretched	3
4	丁	Depicts the top view of a nail	2
5	弓	Depicts the curved shape of a bow	3
6	耳	Depicts the shape of a human ear	6
7	火	Depicts flames rising upward	4
8	户	Depicts a single-leaf door (half of a double-leaf door)	4
9	口	Depicts a human mouth, used for speaking and eating	3
10	马	Depicts a horse’s head, mane, tail, and four legs	3
11	皿	Depicts a vessel used for serving food	5
12	门	Depicts two door leaves joined together	3
13	木	Depicts a tree with branches above and roots below	4
14	目	Depicts a human eye with the pupil marked	5
15	牛	Depicts a cow’s horns, head, body, and tail	4
16	鸟	Depicts a long-tailed bird with feet	5
17	犬	Depicts a dog in profile	4
18	人	Depicts the form of arms and legs of a standing person	2
19	日	Depicts the sun as a circle enclosing a horizontal stroke	4
20	手	Depicts the shape of a clenched hand	4
21	山	Depicts a high form with rocks; peaks rising from the ground	3
22	丝	Depicts two twisted strands of silk thread	5
23	水	Depicts multiple streams flowing together	4
24	它	Depicts a long creature with a curved, drooping tail	5
25	土	Horizontal strokes depict the ground; vertical stroke depicts growth emerging from it	3
26	兔	Depicts a crouching animal with its tail behind	8
27	牙	Depicts upper and lower teeth interlocking	4
28	羊	Depicts a sheep’s head, horns, legs, and tail	6
29	鱼	Depicts a fish; its tail resembles a swallow’s tail	8
30	穴	Depicts an earthen chamber or cave opening	5
31	竹	Depicts the plant with drooping leaves	6
32	自	Depicts the shape of a human nose	6

All characters are in simplified Chinese. Characters were numbered by pinyin alphabetical order.

**Table 2:** Categorization of characters.

Human		Nature		Animal		Manmade	
人 (person)	口 (mouth)	山 (mountain)	土 (soil)	马 (horse)	鸟 (bird)	车 (car)	门 (door)
牙 (tooth)	耳 (ear)	穴 (hole)	日 (sun)	鱼 (fish)	犬 (dog)	弓 (bow)	册 (book)
自 (nose)	大 (big)	水 (water)	火 (fire)	兔 (rabbit)	它 (snake)	皿 (dish)	户 (house)
目 (eye)	手 (hand)	竹 (bamboo)	木 (wood)	牛 (cow)	羊 (sheep)	丁 (nail)	丝 (silk)

**Table 3:** Latin square counterbalancing of condition order and character-to-condition assignment.

	Character Number			
Participant Group	1–8	9–16	17–24	25–32
Group 1 (ID: 1/5/9/13/17/21/25/29)	ND	AD	CD	ACD
Group 2 (ID: 2/6/10/14/18/22/26/30)	AD	CD	ACD	ND
Group 3 (ID: 3/7/11/15/19/23/27/31)	CD	ACD	ND	AD
Group 4 (ID: 4/8/12/16/20/24/28/32)	ACD	ND	AD	CD

Each row represents one participant group. Column headers indicate the numbered IDs of the 32 Chinese pictographic characters used in the study. The assignment was rotated across groups so that each character set appeared under each condition equally often. Column order (left to right) also reflects the learning sequence (Module 1 to Module 4).

**Table 4:** Interview themes and participant descriptive excerpts.

Final Theme	Category	Example Statement
Visual Appeal and Novelty	Visual Monotony	*“This material does not have any design; it’s really boring. (ND)”* (P1)
		*“It’s just the basic and traditional one. This is too plain. (ND)”* (P6, 10, 13)
	Anthropomorphism without Appealing	*“The colors are dull or absent, making the design unpleasant and even uncomfortable. (AD)”* (P1, P3)
		*“But this one only has eyes, and only in black color. It is not attractive. (AD)”* (P13)
	Playful and Eye-Catching	*“Animal-type like the sheep one, are fun and engaging. (CD)”* (P8, 29)
		*“They are attractive, colorful, and can capture the learner’s attention while explaining the function well. (ACD)”* (P5, 9, 10, 31)
Clarity of Design	Clarity and Simplicity	*“This is also good because it’s clear, it’s visible, and clearly seen. (ND)”* (P30)
		*“I think this Chinese pictographic characters is very simple and professional. (ND)”* (P22)
	Complex and Confusing	*“The design feels thick, complex, and difficult to read, making it hard to follow. (AD)”* (P20, 21)
		*“It was a little bit confusing, like it was relating to some living material. (AD)”* (P31)
	Recognition-Hindering Elements	*“Fire is direct. This one is completely not helpful—this ‘house’—but my brain reshaped it differently. (CD)”* (P20)
		*“I find the strokes of the character hard to see clearly because they are too thick. (CD)”* (P22)
Comprehensibility of Design	Lack of Aids for Understanding	*“Very difficult to understand and offers no clue or aid for learning, making it unhelpful for memorization. (ND)”* (P3, 5, 10, 32)
		*“Does not express the meaning clearly or provide elements that help comprehension. (ND & AD)”* (P4, 16)
	Symbolic Cues for Comprehension	*“When design elements match the meaning, they help connect concepts, aid memory, and make learning quicker. (CD & ACD)”* (P7, 14, 26, 27, 30)
		*“It’s attractive and informative because we can understand—it gives a kind of explanation. (ACD)”* (P19, 24)
Impact of Eye Design	Eye Features: Gains and Losses	*“Eye designs can feel distracting or too human-like, making it harder to focus or understand. (AD & ACD)”* (P6, 12, 17)
		*“Eyes in several pictures (like animals) help. (ACD)”* (P4)
		*“Without eyes, characters lose some personality or story element. (CD)”* (P18, 27)
		*“Eyes make characters appear lively and engaging and sometimes help illustrate meaning. (ACD)”* (P13, 30)

## Data Availability

The data presented in this study are available within the article. Additional raw data (e.g., interview transcripts) and processing codes are available on request from Jiaqi Wang (jiaqiwang@sjtu.edu.cn). These data are not publicly available due to privacy and ethical restrictions.

## Notes

1 https://webflow.com/ (accessed on 28 April 2026).

2 https://github.com/raphaelvallat/pingouin (accessed on 28 April 2026).

3 https://plotnine.org/ (accessed on 28 April 2026).
